# The Effect of a Low-Carbohydrate High-Fat Diet on Laboratory Parameters in Women with Lipedema in Comparison to Overweight/Obese Women

**DOI:** 10.3390/nu15112619

**Published:** 2023-06-02

**Authors:** Małgorzata Jeziorek, Andrzej Szuba, Monika Sowicz, Agnieszka Adaszyńska, Krzysztof Kujawa, Angelika Chachaj

**Affiliations:** 1 Department of Dietetics and Bromatology, Faculty of Pharmacy, Wroclaw Medical University, 50-367 Wroclaw, Poland; malgorzata.jeziorek@umw.edu.pl; 2 Department of Angiology, Hypertension and Diabetology, Wroclaw Medical University, 50-367 Wroclaw, Poland; andrzej.szuba@umw.edu.pl (A.S.); monika.sowicz@wp.pl (M.S.); agnieszkarogala87@gmail.com (A.A.); 3 Statistical Analysis Center, Wroclaw Medical University, 50-367 Wroclaw, Poland

**Keywords:** lipedema, obesity, low-carbohydrate high-fat diet, blood parameters

## Abstract

The aim of this study was to evaluate alterations in blood parameters after a low-carbohydrate high-fat (LCHF) diet in women with lipedema in comparison to overweight or obese women. A total of 115 women were classified into two groups: the lipedema group and the overweight/obesity group. Both study groups followed the caloric-restricted LCHF diet for 7 months. A total of 48 women completed the study. A reduction in body weight was observed in both study groups. A significant decrease in triglycerides and an increase in HDL-C concentrations were observed in both study groups. Despite the increase in the concentration of LDL-C observed in the lipedema group, changes in LDL-C differed between individual patients. Improvements in liver parameters, glucose tolerance, and a decrease in fasting insulin levels were observed, although they were less pronounced in the lipedema group than in the overweight/obesity group. Kidney and thyroid functions were similar before and after the LCHF diet in both groups. The LCHF diet may be a valuable nutritional strategy for lipedema and overweight/obese women, with a beneficial effect on weight, glucose profile, liver function, the concentration of triglycerides, and HDL-C and with no effect on kidney and thyroid function.

## 1. Introduction

Lipedema is a chronic adipose tissue disorder that typically affects the lower extremities, excluding feet. In some cases, it may also affect the arms. Clinical symptoms of lipedema include the enlargement of fat tissue within the legs, a tendency to easy bruising, and spontaneous or pressure-induced pain of adipose tissue. Marked disproportion between the upper and lower body is one of the visual symptoms of lipedema [1,2]. Lipedema is a common disorder almost exclusively found in women. It may affect approximately 11% of adult women worldwide [3,4]. The etiology of the disease is still unknown but a few factors seem to be involved in the pathogenesis, including genetic factors and hormonal changes [5,6]. It develops during hormonal changes including pregnancy, childbirth, or menopause [4,7]. The progression of lipedema also usually begins when the body weight increases [8]. Obesity often co-occurs with lipedema [1,9,10]. In our previous study, we demonstrated that 78% of patients with lipedema were overweight or obese based on the body mass index (BMI) [11]. Management of existing overweight or obesity is essential in lipedema treatment [1,2,6,7,11]. Moreover, lipedema is a disorder characterized by inflammation in the affected adipose tissue [4,12]. Notably, inflammation and angiogenesis may occur independently of obesity in lipedema [4]. Therefore, it seems that the best treatment for lipedema patients is a calorie-reduced and anti-inflammatory diet [13]. Some authors suggested that the ketogenic diet can be effective in lipedema treatment [12,14,15]. According to relevant data, nutritional strategies based on carbohydrates limitations can also be recommended as an effective dietary treatment for overweight/obese patients who need significant and substantial weight loss [16,17,18]. We have already shown that a low-carbohydrate high-fat diet (LCHF; 6.1% energy from carbohydrates; 72.3% energy from fat) was more effective than a low-glycemic diet with medium carbohydrate and fat content (MCMF; 39.1% energy from both carbohydrates and fat) in weight management and decreasing of fat tissue from the legs in lipedema patients [11].

A diet with carbohydrate restrictions can not only help with weight loss but also improve the control of blood glucose and lipid parameters in overweight or obese patients [16,17,18]. Recent data suggested that patients with prediabetes or type 2 diabetes who follow a low-carbohydrate diet may be able to improve glycemic control [17,19,20]. The low carbohydrate strategy is associated with a significant improvement in non-alcoholic fatty liver disease (NAFLD) [21]. This diet, besides helping patients to lose weight and reversing the signs of metabolic syndrome, can also reduce inflammation [12]. However, there is no study investigating an association of a low-carbohydrate high-fat diet with blood results, including glucose and lipid profile, liver enzymes, kidney function, and thyroid function, in a long-term period in patients with lipedema. 

The aim of our study was to evaluate the alterations in liver enzymes, glucose and lipid profile, kidney function, and thyroid function after dietary intervention with a low-carbohydrate high-fat (LCHF) diet in women with lipedema in comparison to overweight or obese women. 

## 2. Materials and Methods

### 2.1. Study Design and Subjects

**Figure 1:**
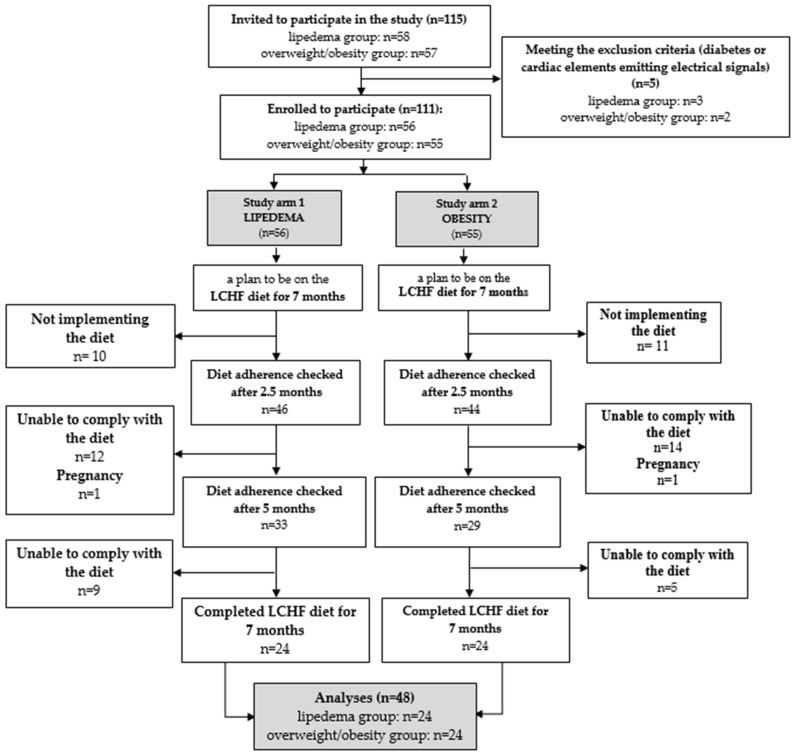
Scheme of study participant selection.

A total of 115 women were invited to participate in the case-control study. The study group consisted of patients from the Angiology Outpatient Clinic at Wroclaw Medical University in Poland with the diagnosis of lipedema [2]. The control volunteer group was composed of women with overweight or obesity (body mass index, BMI > 25 kg/m^2^) who were not affected by lipedema. In the period between January 2021 and May 2022, we consecutively enrolled all the participants in the study. The exclusion criteria for all the study subjects included: pregnancy, breastfeeding, period of 6 months after pregnancy, diagnosis of lymphedema, edema in the course of chronic vein insufficiency or heart failure, diabetes mellitus, kidney or liver failure, hormonally unbalanced thyroid disease, cancer, implanted cardiac devices (cardiac pacemaker, implantable cardioverter-defibrillator, resynchronization therapy) or metal implants. After meeting the inclusion and exclusion criteria, we finally enrolled in the study 58 women with lipedema (with normal weight or with overweight/obesity) and 57 women with overweight/obesity. Figure 1 demonstrates the scheme of study participant selection. A total of 48 female patients completed the 7 months LCHF diet (24 women in the lipedema group and 24 women in the overweight/obesity group). The median age of the participants who completed the study was 39.0 years in the lipedema group and 49.0 years in the overweight/obesity group. Patients in the lipedema group were classified into four clinical stages and five types [6,22,23]. The study was conducted according to the guidelines laid down in the Declaration of Helsinki, and all the procedures involving human patients were approved by the Bioethics Committee at Wroclaw Medical University, Poland (KB-690/2017). Written informed consent was obtained from all the participants.

### 2.2. Measurement of Anthropometric Parameters

A TANITA HR-001 growth meter (Tanita, Tokyo, Japan) was used for height measurement. A TANITA MC-780MA (Tanita, Tokyo, Japan) was used for weight measurement. Body Mass Index (BMI) was calculated as the ratio of body weight [kg] to height [m] squared. Waist, hip, thigh, calf, and ankle circumferences were measured with a standard tape measure to the nearest 1 cm. The mean value of the waist–hip ratio (WHR) was calculated as the ratio of waist to hip circumference.

### 2.3. Blood Samples

Blood samples were collected after 12 h of fasting at baseline and at the end of the intervention (after 7 months of LCHF diet) in both groups. The parameters measured included: bilirubin, aspartate aminotransferase (ASPAT), alanine transaminase (ALAT), gamma-glutamyl transpeptidase (GGTP), alkaline phosphatase (ALP), total cholesterol (TC), low-density lipoprotein cholesterol (LDL-C), high-density lipoprotein cholesterol (HDL-C), triglycerides (TG), creatine, glomerular filtration rate (GFR), uric acid, thyroid stimulating hormone (TSH), glycated hemoglobin (HbA1c), glucose and insulin level. The three-point OGTT (oral glucose tolerance test) with 75 g of glucose solution was performed. Homeostatic Model Assessment for Insulin Resistance (HOMA-IR) was calculated as the ratio of glucose [mg/dl] and insulin [uU/mL] in fasting to values equal 405 [24].

### 2.4. Dietary Intervention

All the involved participants received the personalized caloric-restricted low-carbohydrate high-fat diet, based on the patient’s preferences. They received individual 7-day meal plans to repeat for 7 months with recipes and a shopping list. The dietary plans were created by a professional dietician using DietetykPro software (DietetykPro, Wroclaw, Poland). The personalization of the dietary plans aimed to increase compliance with the diets. Additionally, each patient received detailed dietary recommendations that facilitated adherence to the dietary plan. Dietary plans were based on individual calculated total energy requirements [resting metabolic rate (RMR) × physical activity level (PAL)]. RMR was measured with indirect calorimetry by a Fitmate device (Cosmed, Rome, Italy). RMR was measured at baseline, in the fasting state, following standard procedures [25]. PAL was selected individually for each patient [26]. The energy of each diet was calculated as 75–85% of total energy requirements depending on body weight. The total diet’s energy was divided into four meals including three main dishes and one snack per day. The energy and nutritional status of diets was calculated based on the Polish Tables of Nutritional Value of Products and Dishes [27] and using the USDA (U.S. Department of Agriculture) food products database [28]. 

The LCHF diet was based on animal products such as: lean meat (chicken and turkey), oily marine fish (salmon, herring, sardine, and mackerel), eggs, and full-fat dairy (Greek yogurt, cream, quark, and cheese). The LCHF diet included plenty of plant-based products such as olive and canola oil, avocado, nuts (walnuts, hazelnuts, and almonds), seeds (sunflower, pumpkin, and flaxseed), non-starchy vegetables (zucchini, cucumber, lettuce, tomato, broccoli, cabbage, radish, etc.), berry fruits, and herbs. The interventional diet was planned to provide mostly mono- (MUFA) and polyunsaturated fatty acids (PUFA), antioxidants, and other anti-inflammatory nutrients. The diet was reduced in saturated fatty acids (SFA) and processed foods. All participants were instructed to drink water, natural coffee, and tea. They were not allowed to drink anything else. We designed a low-carbohydrate high-fat diet based on the typical foods of the Mediterranean diet which could be adapted for patients with lipedema. 

All participants were encouraged to maintain a light physical activity level throughout the intervention (20–30 min of light walking a day). 

By conducting a 24 h nutritional interview in regular monthly checks through calls or personal visits and after specific intervals during the study (at 2.5 and 5 months), we were able to gather valuable information on participants’ dietary adherence. These measures helped identify common nutritional mistakes, such as consuming carbohydrate-rich products, skipping meals, or imposing unnecessary fat restrictions. Excluding participants who did not adhere to the prescribed diet was an approach to ensure the integrity of the study and maintain the consistency of the intervention across the study group. Additionally, we controlled the weight and circumference measurements after 2.5 and 5 months (control points).

### 2.5. Statistical Analysis

Results are presented as mean values ± standard deviation or median and quartiles Q1 and Q3 when the data distribution was normal or non-normal, respectively. The conformity of the distribution in a given variable to the normal distribution was verified using the Shapiro–Wilk test. If the distribution was statistically significantly different from normal, non-parametric tests were applied (Mann–Whitney U for independent samples and Wilcoxon test for repeated measurements). Otherwise, *t*-tests were performed. The differences in BMI categories between groups were assessed by the Chi-squared test. To compare the differences between baseline and final anthropometric and blood values, three types of tests were used: *t*-test, Wilcoxon, and Mann–Whitney *U*-tests (depending on the results of the test checking normality of distribution and the test checking the homogeneity of variation). The McNemar test was used to determine if there were differences in the frequency of disturbances in repeated measurements. Results for all analyses were considered statistically significant when *p* < 0.05. STATISTICA v 13.0 from StatSoft Inc. (StatSoft Inc., Tulsa, OK, USA) was used for statistical analysis of the results.

## 3. Results

### 3.1. Characteristics of Study Groups 

The patients in the overweight/obesity group had significantly larger waists than the lipedema group at baseline (106.7 ± 10.6 cm vs. 98.0 ± 12.9 cm; *t*-test: *t* = 2.26, df = 46, *p* = 0.013). This proves that overweight or obese women participating in this study were mostly characterized by visceral obesity. The mean WHR was statistically lower in the lipedema group, which is typical for these patients due to the disproportionate lower extremity fat relative to upper body fat. The mean BMI did not significantly differ between groups (*t*-test: *t* = 1.47, df = 46, *p* = 0.15), which was an important study inclusion criterion. The participants of each BMI class were similar in both groups, except the obesity group did not include women with a BMI < 25 kg/m^2^. We did not report a significant difference between age, weight, hips, and legs circumferences at baseline, except the left calf.

The participants in both groups were not metabolically different at baseline. However, the mean LDL-C level (≥115 mg/dL) indicated hypercholesterolemia in both groups at the beginning of the study. The patients in the overweight/obesity group had a significantly higher LDL-C concentration (145.9 ± 53.5 mg/dL) than the lipedema group (118.0 ± 28.6 mg/dL). The clinical characteristics of the study groups are presented in Table 1. 

The majority of women in the lipedema group were in the second stage of the disease (54.2%). The most common type of lipedema was type 3 (70.8%). The characteristics and prevalence of stages and types of lipedema are summarized in Table 2.

### 3.2. Anthropometric Measurements

Body weight and values of all anthropometric parameters decreased significantly in both groups after 7 months of diet. The exception was a lack of differences between ankle circumferences in the overweight/obesity group. We reported a significantly greater decrease in calf and ankle circumference in the lipedema group compared to the obesity group. The changes in anthropometric measurements after dietary intervention are presented in Table 3.

### 3.3. Diet Composition

Each participant received an individually prepared diet to repeat during the time of the study intervention. Individualization of the dietary plans increased compliance with the diet over the 7 months. The algorithm of individual calculation of total energy requirements and food products included in the diets of both groups was similar. Therefore, we did not report any significant differences between the energy values of interventional diets. The structure of total energy of diets in the lipedema group included 6.9 ± 2.8% of total carbohydrates, 71.1% (70.4–74.4) of fat, and 21.0 ± 2.7% of protein. In the overweight/obesity group, the content of carbohydrates was 7.6 ± 1.3% of total carbohydrates, 70.7% (68.7–72.3) of fat, and 21.7 ± 1.9% of protein. The detailed diet’s energy composition in both groups is presented in Table 4.

### 3.4. Laboratory Blood Tests Results

**Figure 2:**
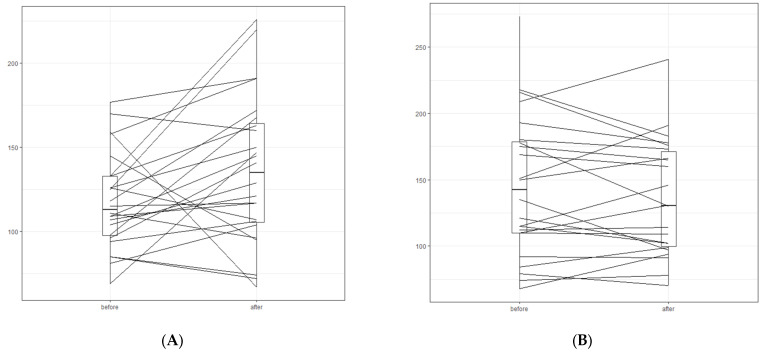
Change in LDL-C concentration [mg/dL] of each participant before and after dietary intervention in the lipedema and the overweight/obesity group. (**A**) lipedema group; (**B**) obesity group.

After the dietary intervention, we did not observe a significant deterioration in most of the blood parameters in either study group, despite the statistically significant increase in the concentration of LDL-C in the lipedema group (an increase of 15.8% from baseline). However, changes in LDL concentrations after the LCHF diet in both study groups differed significantly from patient to patient. Individual responses in LDL-C concentrations to the LCHF diet are shown in Figure 2.

Notably, a statistically significant decrease in triglycerides (12.9% in lipedema vs. 33.2% in obesity) and an increase in HDL-C (9.4% in lipedema vs. 7.0% in obesity) were observed in both study groups. Improvements in liver parameters and glucose tolerance and a decrease in fasting insulin levels were also observed in both study groups, although they were less pronounced in the lipedema group. Kidney and thyroid functions were similar before and after the LCHF diet in both groups. A comparison of laboratory blood test results between the study groups is summarized in Table 5.

The prevalence of the diagnoses of metabolic alterations in the study groups before and after dietary intervention based on the laboratory findings are presented in Table 6. A significant number of women in both study groups had hypercholesterolemia (LDL-C ≥ 115 mg/dL) at baseline (Table 6). The prevalence of other metabolic alterations such as impaired glucose fasting (IGF), impaired glucose tolerance (IGT), insulin resistance (based on HOMA-IR), hyperuricemia, and hypertriglyceridemia at baseline were more often present in the overweight/obesity group than in the lipedema group (Table 6). However, due to the small sample size, we do not discuss these observations.

The frequency of diagnosis of hypertriglyceridemia (TG > 150 mg/dL) significantly decreased after dietary intervention in the overweight/obesity group. The occurrence of hypertriglyceridemia after dietary intervention was comparable in both groups (Table 6).

## 4. Discussions

The dietary intervention was designed to assess the impact of 7 months of the LCHF diet on body weight and blood parameters, including glucose and lipid profiles, liver enzymes, kidney and thyroid function, and uric acid level in both lipedema and obese patients. By analyzing these outcomes, we aimed to provide a comprehensive understanding of the efficacy and safety of the LCHF diet in these patient populations. 

One noteworthy observation is that the LCHF diet did not worsen most of the blood parameters in either study group. We only observed minor alterations in LDL-C concentrations in the lipedema group. However, it is worth noting that the changes in LDL-C concentrations were not consistent across all participants in the lipedema group. Similarly, LDL-C changes were non-uniform across the overweight/obesity group, making it difficult to draw definitive conclusions regarding the effect of the LCHF diet on LDL-C concentrations. It may be presumed that the impact of the LCHF diet on blood lipid concentrations is complex, because it may be influenced by multiple factors, including genetics, hormones, and metabolic factors [29]. The improvement in TG and HDL-C concentrations were more pronounced in the overweight/obesity group than the lipedema group; however, the overweight/obesity group had a higher prevalence of hypertriglyceridemia at baseline. The reduction in TG concentration was more substantial in the overweight/obesity group with a 33.2% decrease compared to a 12.9% decrease in the lipedema group. Additionally, there was a 9.4% increase in HDL-C concentration in the lipedema group, which may also contribute to a reduction in cardiovascular disease (CVD) risk [29]. It is important to note that the increase in LDL-C and HDL-C concentrations observed in both our study groups and in the study by Tzenios et al. [29] after following a very low carbohydrate ketogenic diet may not be clinically relevant, as was suggested by the latter study. However, in the study by Tzenios et al. [29], changes in TG concentrations were not reported, which is not line with our results. Burén et al. [30] observed that the LCHF diet, which is rich in saturated fatty acids and low in dietary fiber induced significant changes in blood lipids. Specifically, after four weeks of following the LCHF diet, they observed higher concentrations of LDL-C compared to a control diet. However, they also noted that individual responses to the LCHF diet varied within the group. Additionally, the LCHF diet induced significant changes in TG levels, which is consistent with the findings of our study. Unlike our results, Burén et al. [30] observed a decrease in HDL-C levels after following the LCHF diet. They concluded that the LCHF diet induced profound and widespread alterations in blood lipids.

It is promising to see that the LCHF diet did not have a negative impact on liver function and even showed slight improvements in liver parameters. The results of this study are consistent with other studies, such as those conducted by Cunha et al. [31], D’Abbondanza et al. [21], and Rinaldi et al. [32], which suggest that a very-low-carbohydrate high-fat diet may be beneficial for patients with NAFLD. The metabolic shift from carbohydrates to triglycerides as the main energy source may contribute to the reduction in liver fat accumulation, especially in overweight/obese patients with increased visceral adipose tissue. Therefore, reducing visceral fat may be a key factor in the resolution of NAFLD. However, more research is needed to confirm the effectiveness of the LCHF diet in NAFLD treatment.

It is also important to note that some studies have reported no significant changes in kidney function parameters after following a very-low-carbohydrate ketogenic diet (VLCKD) [31,33]. It is still important to monitor kidney function regularly, especially in individuals with pre-existing kidney conditions. We did not report alterations in kidney parameters, such as creatinine and GFR, in lipedema. We observed significant changes in both parameters in obesity, but we did not consider it as a disadvantage of the LCHF diet. In the study by Joshi et al. [33], the authors concluded that a low-carbohydrate diet could be safe even in patients with kidney disease. 

Regarding uric acid concentrations, some authors reported lower concentrations of uric acid in patients undergoing VLCKD [31]. Our results are not in line with those, and we demonstrated a lack of change in uric acid concentrations in both study groups. Rinaldi et al. [32] did not observe changes in the concentration of uric acid after 8 weeks of VLCKD on NAFLD in the overweight/obese patients. It is important to note that individual responses to dietary interventions can vary, and there may be several factors that influence changes in uric acid concentrations, including genetics, medication use, and other lifestyle factors. Therefore, it is possible that the differences in findings between different studies could be due to variations in the study populations, dietary protocols, and other factors. It is also possible that longer-term follow-up may be needed to fully evaluate the effects of LCHF diets on uric acid levels.

In this study, TSH was not affected by the LCHF diet in the overweight/obesity group, and it was decreased in the lipedema group. However, the observed decrease was not clinically relevant. The study conducted by Rinaldi et al. [32] showed similar results regarding to TSH after 8 weeks of VLCKD. It is important to note that changes in TSH levels should always be interpreted in the context of thyroid function and individual patient characteristics. While the observed decrease in TSH in the lipedema group may not be clinically relevant in this study, it may be important for individuals considering the LCHF diet to monitor their thyroid function.

The relationship between low carbohydrate diets and glucose and insulin control has been studied in both non-non-diabetes and diabetes patients [16,19,20,34,35]. In this study, the LCHF diet resulted in a decrease in fasting insulin levels without a change in fasting glucose levels in obese participants, and it also improved HOMA-IR and glucose tolerance. However, the study did not evaluate changes in glucose–insulin functioning in lipedema patients. These findings are in accordance with previous studies such as Michalczyk et al. [16], which showed a lower concentration of fasting insulin and glucose concentration and a decrease in HOMA-IR after 12 weeks of a low-calorie ketogenic diet compared to a control group. Additionally, Choi et al. [17] found in a meta-analysis of 14 studies that a ketogenic diet for 3 to 12 months was more effective for glycemic control, as indicated by a significant reduction in HbA1c and HOMA values for diabetic patients. The meta-analysis also showed that a ketogenic diet for 4 days up to 2 years led to improved lipid profiles for diabetic patients, such as lower triglyceride and higher HDL concentrations, whereas for nondiabetic patients, there was an increase in total cholesterol and LDL concentrations. The authors of the meta-analysis noted that lowered postprandial glucose and enhanced insulin sensitivity improved glycemic profiles in the studies analyzed. While Tzenios et al. [29] did not observe changes in fasting glucose after 140 days of VLCKD, they concluded that increased consumption of monounsaturated fatty acids may improve insulin sensitivity and glycemic control.

The study by Sørlie et al. [14] adds to the growing body of research on the potential benefits of LCHF diets for patients with lipedema. The reduction in TG concentration and HbA1c rate, along with the lack of negative effects on other metabolic markers, suggests that the LCHF diet may be a promising treatment option for lipedema patients. Additionally, the improvement in quality of life reported by the participants indicates that this dietary intervention may have broader benefits beyond metabolic health.

The present study adds to the existing literature supporting the idea that the use of LCHF diets has the potential to improve metabolic outcomes in lipedema and overweight/obese patients. The observed improvements in weight, BMI, TG, HDL-C, liver parameters, glucose tolerance, and a decrease in fasting insulin levels suggest that this dietary approach may have a positive impact on the management of these conditions. Importantly, the lack of adverse effects on kidney and thyroid functioning highlights the safety of this dietary intervention. However, further research is needed to investigate the long-term effects of LCHF diets in these populations and to determine optimal dietary approaches for individual patients. Overall, this study provides important insights into the potential benefits of LCHF diets and supports the need for further investigation in this area.

By having a relatively long duration of dietary intervention, the study can provide more reliable information on the effectiveness and safety of LCHF diets in the treatment of lipedema. Additionally, the inclusion of the overweight/obesity group allows for a better comparison between the two study groups and provides a more robust interpretation of the results. The individualization of dietary plans among participants also allows for a more tailored approach, which can potentially increase adherence to the diet and improve the outcomes of the intervention.

Some limitations of the study should be noted. First, the sample size was relatively small, which may limit the generalizability of the findings. A larger sample size would provide more statistical power and increase the ability to detect minor effects. Second, the study did not measure significantly long-term outcomes beyond the 7-month intervention period (such as more than 12 months). Therefore, the long-term effects of LCHF diets on metabolic health and weight management in lipedema patients and overweight or obese women remain unclear.

## 5. Conclusions

The study suggests that the LCHF diet may be a valuable nutritional strategy for both lipedema and obese patients, with a beneficial effect on weight, glucose profile, liver function, the concentration of triglycerides, and HDL-C, and with no effect on kidney and thyroid functions. The effect of the LCHF diet on LDL-C varied in different patients. Therefore, it may be advisable to monitor this parameter during the LCHF diet, and in some patients, it may be beneficial to administer a lipid-lowering drug at least for the time of the use of the diet.

## Figures and Tables

**Table 1:** Clinical characteristics of the study groups at baseline.

Parameter	Lipedema Group (*n* = 24) mean ± SD/Me (Q1, Q3)	Overweight/Obesity Group (*n* = 24) mean ± SD/Me (Q1, Q3)	*t/Z*	*p*-Values
Age [years]	39.0 (34.0, 62.0)	49.0 (41.5, 59.0)	1.28	NS **
Height [cm]	165.2 ± 7.5	164.2 ± 5.9	−0.5	NS *
Weight [kg]	86.1 ± 17.8	92.0 ± 14.9	1.25	NS *
BMI [kg/m^2^]	31.6 ± 6.4	34.0 ± 4.6	1.47	NS *
<25 kg/m^2^	16.7% (*n* = 4)	*n* = 0		NS
25.0–29.9 kg/m^2^	16.7% (*n* = 4)	25.0% (*n* = 6)		NS
30.0–34.9 kg/m^2^	41.7% (*n* = 10)	41.7% (*n* = 10)		NS
35.0–39.9 kg/m^2^	8.3% (*n* = 2)	25.0% (*n* = 6)		NS
>40 kg/m^2^	16.7% (*n* = 4)	8.3% (*n* = 2)		NS
Waist [cm]	98.0 ± 12.9	106.7 ± 10.6	2.57	0.013 *
Hips [cm]	115.1 ± 12.0	114.6 ± 8.6	0.17	NS *
WHR	0.85 ± 0.1	0.93 ± 0.1	−4.2	0.0001 *
Left thigh [cm]	65.1 ± 7.5	64.9 ± 5.6	0.13	NS *
Right thigh [cm]	65.1 ± 7.4	64.9 ± 5.8	0.1	NS *
Left calf [cm]	44.3 (41.3, 47.5)	42.0 (40.5, 44.0)	1.97	0.048 **
Right calf [cm]	45.5 (40.3, 47.8)	42.3 (39.8, 44.5)	1.54	NS **
Left ankle [cm]	24.8 (23.3, 26.0)	23.0 (22.3, 24.8)	1.92	NS **
Right ankle [cm]	24.0 (23.0, 26.0)	23.5 (22.5, 24.8)	1.39	NS **
RMR [kcal]	1651.2 ± 250.8	1676.4 ± 361.2	0.27	NS *
Bilirubin [mg/dL]	0.65 (0.5, 0.85)	0.55 (0.4, 0.65)	2.09	0.034 **
ASPAT [U/L]	21.0 (19.0, 28.5)	25.0 (20.5, 28.0)	1.05	NS **
ALAT [U/L]	19.5 (15.0,25.5)	23.5 (17.5, 29.0)	1.53	NS **
GGTP [U/L]	17.5 (13.0, 22.5)	19.5 (13.5, 26.0)	0.91	NS **
ALP [U/L]	69.6 ± 22.7	67.1 ± 24.0	0.36	NS *
TC [mg/dL]	206.7 ± 40.2	232.3 ± 49.2	1.97	NS *
LDL-C [mg/dL]	118.0 ± 28.6	145.9 ± 53.5	2.25	0.029 *
HDL-C [mg/dL]	67.0 (56.5, 73.0)	64.5 (52.0, 71.0)	0.68	NS **
TG [mg/dL]	83.0 (61.5, 113.5)	118.5 (70.0, 154.5)	1.61	NS **
Creatinine [mg/dL]	0.81 (0.71, 0.83)	0.85 (0.8, 0.92)	2.17	0.03 **
GFR [ml/min/1.73 m^2^]	87.5 ± 15.1	77.2 ± 13.6	2.49	0.016 *
Uric acid [mg/dL]	4.4 (3.9, 5.6)	5.2 (4.3, 6.0)	1.84	NS **
TSH [uIU/mL]	1.6 (1.3,2.2)	1.6 (1.0, 1.9)	0.94	NS **
HbA1c [%]	5.3 ± 0.3	5.5 ± 0.3	2.39	0.021 *
Glucose 0 min [mg/dL]	82.0 (79.0, 88.5)	86.0 (78.5, 94.0)	−0.8	NS **
Glucose 60 min [mg/dL]	125.1 ± 42.3	148.0 ± 47.0	1.71	NS *
Glucose 120 min [mg/dL]	103.5 ± 33.9	119.4 ± 33.5	1.59	NS *
Insulin 0 min [uU/mL]	5.9 (4.7, 8.7)	7.0 (6.1, 9.0)	1.53	NS **
Insulin 60 min [uU/mL]	50.1 (23.1, 81.7)	59.7 (36.9, 92.4)	0.96	NS **
Insulin 120 min [uU/mL]	23.8 (14.7, 49.2)	43.1 (17.5, 101.0)	−1.6	NS **
HOMA-IR	1.2 (0.9, 1.9)	1.6 (1.2, 2.2)	1.34	NS **

* *t*-test, ** U–Mann–Whitney test; df = 46, except RMR, glucose 60 and 120 min—df = 43; *p* < 0.05 statistically significant values. BMI—body mass index; WHR—waist–hip ratio; RMR—resting metabolic rate; ASPAT—aspartate aminotransferase; ALAT—alanine transaminase; GGTP—gamma-glutamyl transpeptidase; ALP—alkaline phosphatase; TC—total cholesterol, LDL-C—low-density lipoprotein cholesterol; HDL-C—high-density lipoprotein cholesterol; TG—triglycerides; GFR—glomerular filtration rate; TSH—thyroid stimulating hormone; HbA1c—glycated hemoglobin; HOMA-IR—Homeostatic Model Assessment for Insulin Resistance.

**Table 2:** Characteristics and prevalence of stages and types of lipedema in the study [23].

Stage	Description	Percentage of Lipedema Stage in the Study (*n* = 24)
1	skin has a smooth texture with subdermal pebble like feel due to underlying loose connective tissue fibrosis	37.5% (*n* = 9)
2	the skin surface is irregular and pitted, and an orange skin phenomenon is present	54.2% (*n* = 13)
3	deformation of legs with larger fat masses	8.3% (*n* = 2)
4	concomitant lymphedema (lipolymphedema)	0
**Type**	**Description**	**Percentage of Lipedema Types in the Study (*n* = 24)**
1	buttocks	0
2	buttocks, hips, and thighs	25.0% (*n* = 6)
3	buttocks, hips, thighs, and calves	70.8% (*n* = 17)
4	arms	33.3% (*n* = 8)
5	calves	4.2% (*n* = 1)

**Table 3:** Comparison of anthropometric measurements before and after dietary intervention in the study groups.

Parameter	Lipedema Group (*n* = 24) Mean ± SD/Me (Q1, Q3)			Overweight/Obesity Group (*n* = 24) Mean ± SD/Me (Q1, Q3)			Differences between Baseline and 7 Months Mean ± SD/Me (Q1, Q3)			
	Baseline	7 Months	*p*-Values	Baseline	7 Months	*p*-Values	Lipedema	Obesity	*t/Z*	*p*-Values
Weight [kg]	86.1 ± 17.8	74.1 ± 12.9	<0.0001 *	90.5 (81.7, 97.5)	77.1 (69.2, 86.5)	<0.0001 **	−11.1 (6.4, 15.9)	−11.8 (10.2, 13.1)	−0.84	NS ***
BMI [kg/m^2^]	31.6 ± 6.4	27.3 ± 5.1	<0.0001 *	33.9 (30.5, 36.3)	28.0 (26.1, 33.1)	<0.0001 **	−3.9 (2.5, 6.2)	−4.3 (3.9,4.6)	−0.96	NS ***
Waist [cm]	98.0 ± 12.9	85.2 ± 11.3	<0.0001 *	106.7 ± 10.6	94.4 ± 12.1	<0.0001 *	−12.8 ± 6.4	−12.4 ± 6.2	0.23	NS *
Hips [cm]	115.1 ± 12.0	105.4 ± 9.2	<0.0001 *	115.0 (109.5, 119.8)	104.8 (101.0, 108.3)	<0.0001 **	−8.8 (6.3, 13.0)	−8.3 (7.0, 11.3)	−0.06	NS ***
WHR	0.84 (0.78, 0.91)	0.79 (0.74, 0.86)	0.0001 **	0.93 ± 0.1	0.9 ± 0.1	0.006 *	−0.04 (0.0, 0.07)	−0.02 (0.001, 0.06)	0.69	NS ***
Left thigh [cm]	65.1 ± 7.5	59.1 ± 5.9	<0.0001 *	64.9 ± 5.6	59.2 ± 5.3	<0.0001 *	−6.0 ± 3.3	−5.7 ± 2.2	0.36	NS*
Right thigh [cm]	65.1 ± 7.3	58.9 ± 6.0	<0.0001 *	64.9 ± 5.8	59.1 ± 5.4	<0.0001 *	−6.2 ± 3.5	−5.8 ± 2.4	0.43	NS *
Left calf [cm]	44.7 ± 5.2	40.7 ± 3.9	<0.0001 *	42.1 ± 3.9	39.5 ± 3.7	<0.0001 *	−4.0 ± 2.0	−2.7 ± 1.2	2.8	0.008 *
Right calf [cm]	44.5 ± 5.3	40.8 ± 4.3	<0.0001 *	42.4 ± 4.1	39.9 ± 4.0	<0.0001 *	−3.8 ± 2.0	−2.4 ± 1.0	2.97	0.005 *
Left ankle [cm]	25.0 ± 2.3	23.7 ± 2.1	<0.0001 *	23.0 (22.3, 24.8)	23.0 (22.3, 24.5)	NS	−1.0 (0.5, 1.8)	0.0 (0.0, 0.5)	3.93	<0.0001 ***
Right ankle [cm]	24.0 (23.0, 26.0)	23.0 (22.3, 24.8)	0.0001 **	23.9 ± 2.5	23.7 ± 2.4	NS*	−1.0 (0.5, 2.0)	−0.2 (0.0, 0.5)	3.31	<0.001 ***

* *t*-test, ** Wilcoxon, *** U–Mann–Whitney test; df = 46; *p* < 0.05 statistically significant values, NS—Not Statistically Significant. Please note that the median of the differences between “before” and “after” do not correspond to the differences between the median of data for “before” and the median of data for “after”, as the first one was calculated using paired data. BMI—body mass index, WHR—waist–hip ratio.

**Table 4:** Diet composition in lipedema and obesity group.

Parameter	Lipedema Group (*n* = 24) Mean ± SD/Me (Q1, Q3)	Overweight/Obesity Group (*n* = 24) Mean ± SD/Me (Q1, Q3)	*p*- Values
Energy value [kcal]	1680.8 ± 132.3	1648.7 ± 123.3	NS *
Total protein [g] Total protein [% kcal]	88.3 ± 14.4 21.0 ± 2.7	89.2 ± 6.5 21.7 ± 1.9	NS * NS *
Total carbohydrates [g] Total carbohydrates [% kcal]	33.9 ± 13.0 6.9 ± 2.8	36.5 ± 7.4 7.6 ± 1.3	NS * NS *
Fiber [g]	8.9 (7.7, 10.8)	8.8 (8.1, 11.9)	NS **
Total fat [g] Total fat [% kcal]	133.1 (124.4, 141.1) 71.1 (70.4, 74.4)	132.1 (120.3, 140.0) 70.7 (68.7, 72.3)	NS ** NS **
SFAs [g]	36.0 ± 7.8	36.9 ± 6.9	NS *
MUFA [g]	55.9 ± 8.5	58.5 ± 8.4	NS *
PUFA [g]	24.6 ± 5.7	27.0 ± 6.8	NS *
*n*-6 to *n*-3 ratio	3.1 (2.2, 4.2)	3.3 (3.0, 3.8)	NS **

* *t*-test, ** U–Mann–Whitney test; NS—Not Statistically Significant. SFAs—saturated fatty acids; MUFA—monounsaturated fatty acids; PUFA—polyunsaturated fatty acids; *n*-6—polyunsaturated fatty acids of the omega-6 family; *n*-3—polyunsaturated fatty acids of the omega-3 family.

**Table 5:** Comparison of laboratory blood test results before and after dietary intervention in the study groups.

Parameter	Lipedema Group (*n* = 24) Mean ± SD/Me (Q1, Q3)			Overweight/Obesity Group (*n* = 24) Mean ± SD/Me (Q1, Q3)			Differences Between Baseline and 7 Months Mean ± SD/Me (Q1, Q3)			
	Baseline	7 Months	*p*-Value	Baseline	7 months	*p*-Value	Lipedema	Obesity	*t/Z*	*p*-Value
Bilirubin [mg/dL]	0.65 (0.5, 0.85)	0.7 (0.5, 0.85)	NS **	0.5 (0.4, 0.7)	0.7 (0.5, 0.9)	0.024 **	0.05 (−0.2, 0.1)	0.2 (−0.3, 0.2)	1.51	NS ***
ASPAT [U/L]	23.3 ± 6.5	22.7 ± 5.6	NS *	25.0 (20.5, 28.0)	19.5 (17.5, 23.0)	0.001 **	−0.6 ± 5.6	−5.5 ± 7.0	−2.57	0.01 ***
ALAT [U/l]	20.8 ± 8.9	20.0 ± 7.0	NS *	23.5 (17.5, 29.0)	19.5 (15.0, 21.0)	0.008 **	−0.8 ± 6.8	−8.5 ± 16.0	−2.04	0.04 ***
GGTP [U/L]	17.5 (13.0, 22.5)	15.0 (13.0, 20.0)	NS **	19.5 (13.5, 26.0)	17.5 (14.0, 21.0)	0.022 **	−0.5 (−1.0, 3.0)	−2.0 (−0.5, 5.5)	−1.27	NS ***
ALP [U/l]	63.5 (52.0, 81.5)	64.0 (56.0, 76.0)	NS **	67.1 ± 24.0	60.3 ± 20.0	0.001 *	−2.0 (−8.5, 26.0)	−5.5 (−0.5, 13.0)	−0.59	NS ***
TC [mg/dL]	206.7 ± 40.2	226.4 ± 54.7	NS *	223.0 (190.5, 268.5)	215.0 (194.0, 249.0)	NS **	19.7 ± 50.6	−31.9 ± 89.4	−2.77	0.005 ***
LDL-C [mg/dL]	118.0 ± 28.6	136.6 ± 44.2	0.046 *	142.5 (110.0, 179.0)	130.5 (99.0, 173.0)	NS **	18.6 ± 43.2	−8.0 (−15.5, 27.0)	−2.29	0.022 ***
HDL-C [mg/dL]	67.8 ± 18.8	74.2 ± 20.3	0.014 *	64.5 (52.0, 71.0)	69.0 (54.0,74.0)	NS **	6.4 ± 12.0	2.5 ± 21.7	−1.3	NS ***
TG [mg/dL]	88.5 ± 35.9	77.1 ± 35.8	0.014 *	122.2 ± 68.8	81.6 ± 36.3	<0.0001 *	−11.5 ± 21.2	−47.4 ± 41.7	−3.77	0.0004 *
Creatine [mg/dL]	0.8 ± 0.2	0.78 ± 0.1	NS *	0.9 ± 0.1	0.8 ± 0.1	0.003 *	−0.03 ± 0.08	−0.06 ± 0.08	−1.1	NS *
GFR [mL/min/1.73 m^2^]	87.5 ± 15.1	85.7 ± 16.0	NS *	77.2 ± 13.6	81.1 ± 11.9	0.049 *	−1.8 ± 9.2	3.9 ± 9.2	2.14	0.037 *
Uric acid [mg/dL]	4.4 (3.9, 5.6)	4.2 (3.4, 4.9)	NS **	5.2 (4.3, 6.0)	4.9 (4.4, 5.7)	NS	−0.2 (−0.2, 0.9)	−0.4 (−0.3, 0.6)	0.23	NS ***
TSH [uIU/mL]	1.6 (1.3, 2.2)	1.3 (1.0, 1.7)	0.009 **	1.6 (0.9, 1.9)	1.4 (78.5, 94.0)	NS	−0.3 (−0.04, 0.6)	−0.05 (−0.1, 0.4)	1.29	NS ***
HbA1c [%]	5.3 (5.1, 5.5)	5.3 (5.1, 5.5)	NS **	5.5 ± 0.3	5.5 ± 0.3	NS *	−0.1 (−0.1, 0.1)	−0.01 ± 0.2	−0.31	NS ***
Glucose 0 min [mg/dL]	85.1 ± 11.9	87.9 ± 9.6	NS *	86.8 ± 10.6	84.4 ± 15.6	NS *	−2.8 ± 12.8	−2.3 ± 11.4	−1.47	NS *
Glucose 60 min [mg/dL]	126.1 ± 39.5	149.8 ± 44.4	NS *	148.0 ± 47.0	125.6 ± 47.8	0.0004 *	−5.2 ± 83.2	−42.4 ± 46.7	−2.44	0.018 *
Glucose 120 min [mg/dL]	100.0 ± 22.6	109.1 ± 25.0	NS *	115.0 (100.0, 154.0)	96.5 (79.5, 117.0)	0.002 **	−1.0 (−29.0, 46.5)	−26.0 (4.0, 51.0)	−1.61	NS ***
Insulin 0 min [uU/mL]	5.9 (4.7, 8.7)	5.8 (4.0, 9.6)	NS **	7.0 (6.1, 9.0)	5.7 (3.8, 8.0)	0.0005 **	−0.6 (−1.3, 2.3)	−1.4 (0.4, 4.0)	−2.01	0.044 ***
Insulin 60 min [uU/mL]	50.1 (23.1, 81.7)	40.3 (27.7, 64.8)	NS **	59.7 (36.9, 92.4)	42.8 (28.0, 70.8)	0.008 **	−8.1 (−15.1, 30.3)	−19.3 (1.8, 48.8)	−1.51	NS ***
Insulin 120 min [uU/mL]	33.3 ± 26.6	36.8 ± 21.7	NS *	43.1 (17.5, 100.9)	31.8 (15.5, 44.8)	0.016 **	−0.7 (−11.6, 17.1)	−14.6 (0.7, 38.9)	−2.01	0.044 ***
HOMA-IR	1.2 (0.9, 1.9)	1.0 (−2.5, 5.0)	NS **	1.6 (1.2, 2.2)	1.1 (0.5, 7.5)	0.0007 **	−0.1 (−0.2, 0.4)	−0.3 (0.1, 0.8)	−2.32	0.02 ***

* *t*-test, ** Wilcoxon test, *** U–Mann–Whitney test; df = 46; *p* < 0.05 statistically significant values, NS—Not Statistically Significant. Please note that the median of the differences between “before” and “after” do not correspond to the differences between the median of data for “before” and the median of data for “after”, as the first one was calculated using paired data. ASPAT—aspartate aminotransferase; ALAT—alanine transaminase; GGTP—gamma-glutamyl transpeptidase; ALP—alkaline phosphatase; TC—total cholesterol, LDL-C—low-density lipoprotein cholesterol; HDL-C—high-density lipoprotein cholesterol; TG—triglycerides; GFR—glomerular filtration rate; TSH—thyroid stimulating hormone; HbA1c—glycated hemoglobin; HOMA-IR—Homeostatic Model Assessment for Insulin Resistance.

**Table 6:** The prevalence of diagnoses of metabolic alterations in the study groups before and after dietary intervention based on the laboratory findings.

Diagnosis	Lipedema Group *n* = 24		*p*-Value	Overweight/Obesity Group *n* = 24		*p*-Value
	Baseline	7 Months		Baseline	7 Months	
Hypercholesterolemia (LDL-C ≥ 115 mg/dL)	50.0% (*n* = 12)	66.7 % (*n* = 16)	NS	68.2% (*n* = 15)	50.0% (*n* = 11)	NS
Hypertriglyceridemia (TG ≥ 150 mg/dL)	3.8 % (*n* = 1)	3.8 % (*n* = 1)	NS	31.8% (*n* = 7)	4.5% (*n* = 1)	0.04
HDL-C < 45 mg/dL	3.8 % (*n* = 1)	3.8 % (*n* = 1)	NS	9.1% (*n* = 2)	4.5% (*n* = 1)	NS
IGF (100–125 mg/dL)	3.8 % (*n* = 1)	11.5% (*n* = 3)	NS	9.1% (*n* = 2)	13.6% (*n* = 3)	NS
IGT (140–199 mg/dL)	7.7% (*n* = 2)	7.7% (*n* = 2)	NS	22.7% (*n* = 5)	18.2% (*n* = 4)	NS
HOMA-IR (>2.5)	7.7% (*n* = 2)	7.7% (*n* = 2)	NS	22.7% (*n* = 5)	13.6% (*n* = 3)	NS
Hyperuricemia (>6 mg/dL)	7.7% (*n* = 2)	3.8 % (*n* = 1)	NS	18.2% (*n* = 4)	22.7% (*n* = 5)	NS
ALAT (>35 U/L)	3.8 % (*n* = 1)	3.8 % (*n* = 1)	NS	18.2% (*n* = 4)	0	NS
ASPAT (>31 U/L)	11.5% (*n* = 3)	11.5% (*n* = 3)	NS	13.6% (*n* = 3)	0	NS

*p* < 0.05 statistically significant values, NS—Not Statistically Significant. LDL-C—low-density lipoprotein cholesterol; TG—triglycerides; HDL-C—high-density lipoprotein cholesterol; IGF—impaired glucose fasting; IGT—impaired glucose tolerance; HOMA-IR—Homeostatic Model Assessment for Insulin Resistance; ASPAT—aspartate aminotransferase; ALAT—alanine transaminase.

## Data Availability

All data used to support the findings of this study are available from the corresponding author upon reasonable request.
